# *Rabx-5* Regulates RAB-5 Early Endosomal Compartments and Synaptic Vesicles in *C. elegans*

**DOI:** 10.1371/journal.pone.0037930

**Published:** 2012-06-04

**Authors:** Sharon B. Sann, Matthew M. Crane, Hang Lu, Yishi Jin

**Affiliations:** 1 Neurobiology Section, Division of Biological Sciences, University of California San Diego, La Jolla, California, United States of America; 2 Interdisciplinary Program in Bioengineering, Georgia Institute of Technology, Atlanta, Georgia, United States of America; 3 Howard Hughes Medical Institute University of California San Diego, La Jolla, California, United States of America; Columbia University, United States of America

## Abstract

Early endosomal membrane compartments are required for the formation and recycling of synaptic vesicles, but how these compartments are regulated is incompletely understood. We performed a forward genetic screen in *C. elegans* for mutations that affect RAB-5 labeled early endosomal compartments in GABAergic motoneurons. Here we report the isolation and characterization of one mutation, *rabx-5*. The *rabx-5* mutation leads to decreased intensity of YFP::RAB-5 in the cell soma but increased intensity in the synaptic and intersynaptic regions of the axon. This effect is due to the bias of the cycling state of RAB-5, and results from a change in the organization of the early endosomal compartment as well as the membrane binding state of RAB-5. Synaptic vesicle accumulation is altered in *rabx-5* mutants, and synaptic transmission from cholinergic neurons is decreased. Early endosomal membrane compartments show disorganization with ageing and *rabx-5* mutant animals age faster. These results suggest that *rabx-5* regulation of RAB-5 compartments is important for maintaining proper synaptic function throughout the lifetime.

## Introduction

Synaptic vesicle formation and neurotransmission require precise regulation of the proteins and lipids of the exocytic and endocytic pathways [1], [2], [3]. Synaptic vesicles contain numerous members of the Rab family of membrane regulators [4], [5], [6] suggesting interactions with multiple endosomal populations during formation, secretion, and recycling.

In the activated GTP-bound state, Rab GTPases associate with specific membrane compartments through their C-terminal prenylation motifs. There, they regulate membrane trafficking by recruiting effector proteins that bind to proteins responsible for budding and fusion [7]. Specific Rab proteins act as “intracellular cargo address labels” [7], [8]. Rab-5 functions mainly in early endosomes, Rab-7 is present in late endosomes, and Rab-11 associates with recycling endosomes [9], [10].

In non-neuronal cells, Rab-5 regulates the fusion of endocytic vesicles to early endosomes as well as the homotypic fusion of early endosomes [11]. It also regulates transport of early endosomes on microtubules [12] and the structure of the endoplasmic reticulum [13].

In neurons, Rab-5 is associated with early endosomes in both axonal and somato-dendritic compartments; however only in the latter compartment does it interact with EEA-1 [14]. Rab-5 regulates the polarized sorting and trafficking of proteins to axonal compartments [15]. Rab-5 is also required for retrograde transport and signaling from axons [16], [17]. Furthermore, Rab-5 has been demonstrated to localize to synaptic vesicles [18], [19] along with other Rabs involved in endocytosis and recycling such as Rab10, Rab11, and Rab14 [6].

Rab-5 is required in *Drosophila* for endosomal integrity during synaptic vesicle recycling [20] as well as maintenance of synaptic vesicle size, with impairment of Rab-5 leading to enlarged synaptic vesicles [21]. Biasing RAB-5 to the GTP-bound state in *C. elegans* leads to enlarged endosome-like compartments in the synaptic terminal with a concomitant decrease in synaptic vesicle numbers [22]. Overexpression of Rab-5 in mammalian neurons leads to reduced size of the synaptic vesicle recycling pool in hippocampal cultured neurons [23]. Impaired Rab-5 decreases the probability of evoked neurotransmitter release whereas overexpression increases efficacy of release [20].

While Rab-5 clearly functions in the maturation and recycling of synaptic vesicles, little is known about its regulation in neurons, particularly at the synaptic terminal. To identify proteins that may be involved in neuronal RAB-5 regulation, we conducted a genetic screen in *C. elegans* for mutations that disrupt the expression or localization of fluorescently labeled RAB-5 in GABAergic motor neurons. We identified RABX-5 as one regulator of neuronal RAB-5. We demonstrate that RABX-5 is the guanine exchange factor for RAB-5 in neurons and describe the effects of *rabx-5* and *rab-5* disruption on endosomal populations, synaptic vesicles, locomotion, and ageing.

## Results

### Screen for regulators of the endocytic pathway in neuronal development

**Figure 1:**
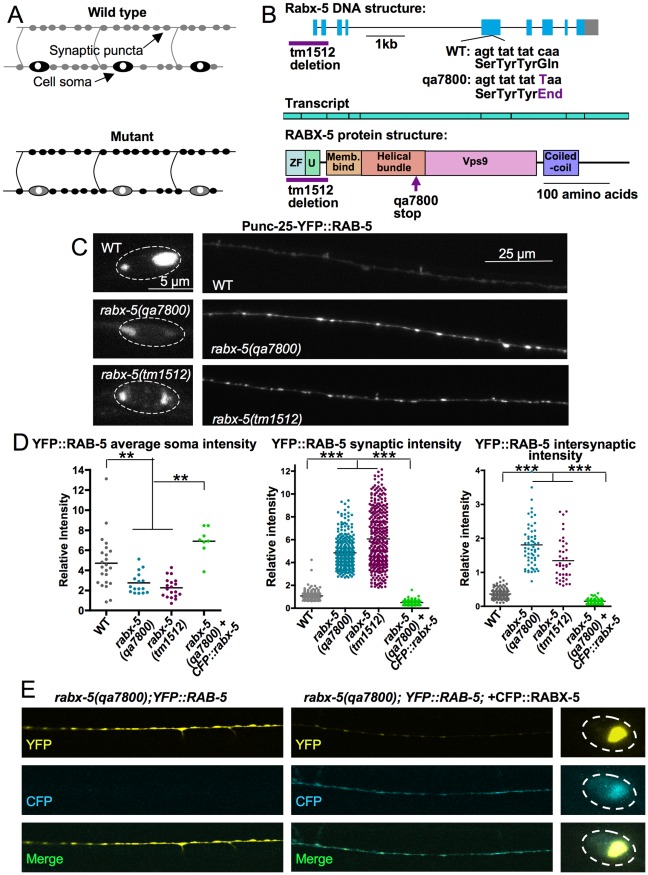
*Rabx-5* mutations alter the RAB-5 organization in GABAergic motor neurons. A) A forward genetic screen was conducted by observing GABAergic motor neurons for changes in localization or intensity of P_unc-25_YFP::RAB-5. Initial screening isolated mutants in which YFP::RAB-5 was decreased in the cell soma. Further inspection determined if YFP::RAB-5 was increased at the synapse. B) Gene, transcript, and protein structure of *rabx-5*. The RABX-5 protein consists of a zinc finger motif (ZF) and a motif interacting with ubiquitin (U) that together regulate association with ubiquitinated endosomal cargo; a membrane binding motif and helical bundle required for association with the endosomal membrane; a Vps9 domain that along with the helical bundle promotes guanine exchange activity; and a coiled-coil region that binds rabaptin-5 and contains a motif for autoinhibition of guanine exchange activity. The *rabx-5(qa7800)* mutation leads to a truncation at the helical bundle. The *rabx-5(tm1512)* mutation deletes the start site and the exons encoding the zinc finger motif and the motif interacting with ubiquitn. C) P_unc-25_YFP::RAB-5 protein localization in the soma (left column) and dorsal cord (right column) of GABAergic motor neurons in wild type and mutant animals. D) P_unc-25_YFP::RAB-5 fluorescence intensity was decreased in the soma of mutant animals and increased in the synaptic and intersynaptic regions. This phenotype is recovered with expression of P_unc-25_CFP::RABX-5. Each point represents a single soma, synaptic puncta, or intersynaptic axonal region, respectively. Bar represents the mean. **p<0.01, ***p<0.001. E) P_unc-25_CFP::RABX-5 expression recovers the *rabx-5(qa7800)* YFP::RAB-5 phenotype.

Several distinct endosomal compartments are present in the GABAergic motor neuron synapse of *C. elegans* [22]. The expression pattern of a fluorescently tagged RAB-5 reporter is specifically altered in mutant animals lacking the function of UNC-16, a kinesin adaptor protein [22], [24]. To identify additional genes involved in the endocytic pathway and axonal transport in neuronal development, we conducted a forward genetic screen using a YFP::RAB-5 reporter in GABAergic motor neurons. These neurons are pseudo-unipolar neurons, with their soma located in the ventral nerve cord, and nerve processes elongating along the ventral and dorsal nerve cord, connected by a circumferential commissure. Presynaptic terminals form en passant along the ventral (for VD neurons) and the dorsal (for DD neurons) processes. We have conducted the screen in two ways: a visual inspection and an automated screen. The visual screen was conducted in an *unc-104* mutant background. UNC-104 is a KIF1A kinesin motor protein. Because of transport defects, *unc-104* mutant worms have decreased numbers of synaptic vesicles marked with syntaptobrevin-GFP at the synapse as well as decreased localization of YFP::RAB-5 to the synapse [22]. Furthermore, *unc-104* mutant worms are severely paralyzed, facilitating the examination of the YFP::RAB-5 protein localization phenotype. In this visual screen, 1,824 haploid genomes have been screened, resulting in the isolation of eight mutants with disrupted YFP::RAB-5 protein localization patterns. A parallel screen, based on a recent technological development using microfluidic automated screening [25] was carried out using YFP::RAB-5 in a wildtype background, screening 1500 haploid genomes and isolating nine mutations. Screening identified animals in which YFP::RAB-5 protein localization was decreased within D neuron cell bodies. Further inspection determined whether there were also synaptic changes (Figure 1a).

These mutations were back crossed, behave as recessive, and have been classified into at least eight different genes based on protein localization patterns of YFP::RAB-5 and movement phenotypes. All identified mutations have decreased YFP::RAB-5 fluorescence intensity within the cell body. 1) One mutation (*ju746*), by non-complementation testing, is an allele of *unc-16*, a known regulator of RAB-5 [22], [26] validating the screen. 2) Three alleles (*ju752, qa7807, qa7808*) have normal synaptic fluorescence intensity of YFP::RAB-5 and an uncoordinated phenotype. 3) One allele (*ju755*) has normal synaptic fluorescence pattern, normal movement, and is approximately 5 map units from *unc-104.* 4) Four alleles (*ju747, ju748, ju758)* have normal synaptic fluorescence pattern, are genetically unlinked to *unc-104,* but require an *unc-104* background for the decreased fluorescence intensity of YFP::RAB-5 in the cell body. Non-complementation tests suggest that these alleles are in separate genes. 5) Six alleles (*ju749, ju751, qa7801, qa7802, qa7803, qa7804*) have normal synaptic fluorescence intensity, wild-type movement, are unlinked to *unc-104* and preliminarily fail to complement one another suggesting that they are one gene. 6) One allele (*qa7809*) has a particularly distinct decrease in cell body fluorescence intensity, and no movement phenotype. 7) One allele (*qa7805*) has increased synaptic fluorescence intensity in addition to decreased fluorescence intensity in the cell body and an uncoordinated movement phenotype. 8) A second allele (*qa7800*) also has increased synaptic and decreased cell body fluorescence intensity, but has no gross movement phenotype. This last mutation, *qa7800*, was mapped to *rabx-5,* the *C. elegans* homologue of rabex-5, a guanine exchage factor (GEF) for Rab-5 in other species.

### *Rabx-5* gene and protein structure and mutant phenotype

The *rabx-5* gene in *qa7800* mutant worms possesses a single nucleotide ct change resulting in a premature stop codon in the fifth exon of *rabx-5* (Figure 1b). The first and second exons of *rabx-5* encode a zinc finger motif that has E3 ubiquitin ligase activity in mammalian homologues [27], [28], [29], and the fifth through seventh exons encode a VPS9 domain that catalyzes guanine exchange in homologous proteins of other species [30]. *Rabx-5(tm1512)* is a deletion mutation which covers the first exon and intron, and part of the second exon. We found that the *rabx-5(tm1512)* mutant revealed a similar YFP::RAB-5 protein localization pattern as with *qa7800* and that *rabx-5(tm1512)* failed to complement *qa7800*. These observations suggest that the YFP::RAB-5 defects likely result from total loss of function in *rabx-5*.

*Rabx-5* mutants exhibit decreased protein localization of YFP::RAB-5 in the cell bodies but increased protein localization within the dorsal cord in both synaptic and intersynaptic regions (Figure 1C and 1D). Both phenotypes can be rescued by the injection of a CFP::RABX-5 construct driven by a GABAergic motor neuron promoter (Figure 1D and 1E). Average soma intensity significantly decreased from 4.6±0.4 relative intensity units (IU) in WT to 2.7±0.3 IU in *rabx-5(qa7800)* and 2.2±0.2 IU in *rabx-5(tm1512)* (p<0.01). The rescue construct restored protein localization to 6.9±0.5 IU. Synaptic intensity significantly increased from 1.0±0.01 IU in WT to 4.8±0.07 IU in *rabx-5(qa7800)* and 6.1±0.13 IU in *rabx-5(tm1512)* (p<0.001). Rescue with CFP::RABX-5 restored synaptic intensity to 0.5±0.02 IU. Average intensity of intersynaptic regions significantly increased from 0.36±0.01 IU in WT to 1.8±0.08 IU in *rabx-5(qa7800)* and 1.3±0.09 IU in *rabx-5(tm1512)* (p<0.01) with intensity in rescue animals at 0.14±0.01 IU. These phenotypes were confirmed using a second integrated P_unc-25_YFP::RAB-5 marker (data not shown).

### Effects of RABX-5 interacting proteins and other GEFs

**Figure 2:**
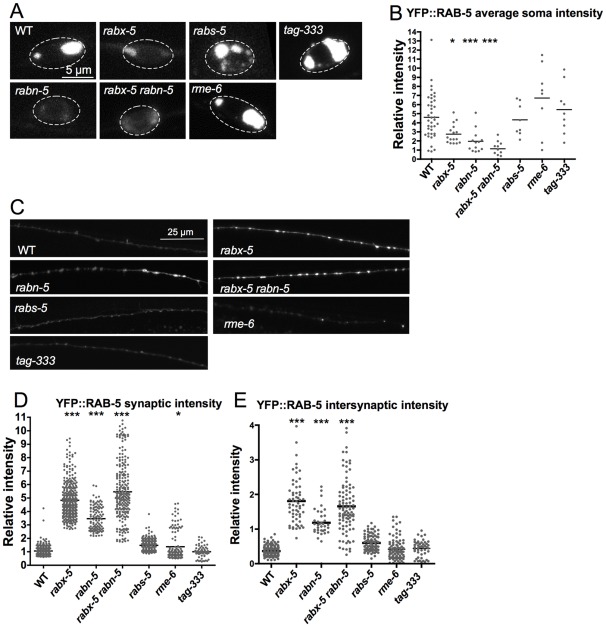
P_unc-25_YFP::RAB-5 localization in mutations of other RAB-5 effectors and VPS9 domain proteins. A) GABAergic motor neuron cell somas expressing YFP::RAB-5 in animals mutant for the RAB-5 effectors, *rabn-5* and *rabs-5,* or mutant for the other VPS9 domain proteins, *tag-333* and *rme-6.* B) Quantification of soma YFP::RAB-5 intensity. C) GABAergic motor neuron dorsal cord in these mutant animals. D) Quantification of synaptic intensity. E) Quantification of intersynaptic intensity. *Rabx-5* works in the same pathway as *rabn-5* but not *rabs-5* to regulate RAB-5. *Rme-6* and *tag-333* do not exhibit the same phenotype as *rabx-5*. Each point represents a single soma, synaptic puncta, or intersynaptic axonal region. Bar represents the mean. *p<0.05, ***p<0.001.

In other systems, the vacuolar sorting protein VPS9 domain has been shown to be the guanine exchange catalytic domain of rabex-5 [30], [31]. Two other *C. elegans* proteins have VPS9 domains as identified by homology. RME-6 is a guanine exchange factor for RAB-5 that regulates clathrin coated vesicles in oocytes and coelomocytes [32]. The uncharacterized protein, TAG-333, is the only other *C. elegans* protein that contains the VPS9 guanine exchange domain. To ask whether these other guanine exchange proteins affect RAB-5 in neurons, we examined YFP::RAB-5 fluorescence intensity in *rme-6(b1014)* and *tag-333(gk431)* mutant animals (Figure 2). *Rme-6* and *tag-333* mutants do not show as dramatic changes in YFP::RAB-5 fluorescence intensity as seen in *rabx-5* mutants; however *rme-6* mutant synaptic intensity (1.5±0.03 IU) is significantly increased compared to WT (1±0.01 IU, p<0.05). From this, we conclude that RABX-5 is the primary guanine exchange protein for RAB-5 in GABA motor neurons, with RME-6 also having a role at synapses.

To further understand the role that RABX-5 plays in regulating RAB-5 labeled endosomes in neurons, we examined other RAB-5 effector proteins. Rabaptin-5 (RABN-5) is a RAB-5 effector that binds to RABX-5 and increases its GDP to GTP exchange activity. It is required for homotypic fusion of RAB-5 early endosomes [33] and may act by preventing the negative autoregulation of RABX-5 [34]. Rabenosyn is a RAB-5 effector that links RAB-5 to the syntaxin binding protein VPS45 [35]. Together these proteins act downstream of RAB-5 to promote endosomal fusion [36]. To determine the role that these proteins play in regulation of RAB-5 endosomes in neurons, we have examined the effects of *rabn-5(tm1555)* and *rabs-5(ok1513)* mutations on YFP::RAB-5 fluorescence pattern (Figure 2).

Fluorescence pattern of YFP::RAB-5 in *rabn-5* mutant animals was similar to that of *rabx-5* mutant animals with a decrease in intensity in cell bodies from 4.6±0.4 IU in WT to 1.9±.3 IU in *rabn-5* mutants and increased synaptic (WT 1±0.01 IU and *rabn-5* 3.5±0.09 IU) and intersynaptic intensity (WT 0.4±0.01 IU and *rabn-5* 1.2±0.07 IU). *Rabx-5 rabn-5* double mutant animals showed a similar decrease in cell body intensity (1.1±0.2 IU) and increase in synaptic (5.5±0.14 IU) and intersynaptic intensity (1.7±08 IU), consistent with *rabn-5* and *rabx-5* working together to promote guanine exchange of RAB-5.

YFP::RAB-5 intensity in *rabs-5* mutants does not show the dramatic changes seen in *rabx-5* mutants, suggesting an independent role. However, *rabs-5* mutant animals do show a change in the pattern of neuronal cell body protein localization of YFP::RAB-5 into increased numbers of discrete compartments. *Rabx-5;rabs-5* double mutants are homozygous lethal.

### Role of guanine exchange in the *rabx-5* phenotype

Mammalian Rabex-5 and Rabaptin-5 function to exchange and stabilize RAB-5 to its GTP-bound state [33]; therefore, loss of function mutations in these proteins likely bias RAB-5 to its GDP bound form. In order to determine if changes in YFP::RAB-5 protein localization in *rabx-5* mutants depend on changes in the nucleotide binding state of RAB-5, we expressed constitutively active YFP::RAB-5(Q78L) or inactive YFP::RAB-5(S33N) [37] in *rabx-5* mutant worms. The RAB-5(Q78L) mutation locks a RAB-5 molecule that has been loaded with GTP into the GTP-bound state. The RAB-5(S33N) mutation biases RAB-5 to its inactive GDP-bound state. If changes in RAB-5 localization depend on its GTP-binding state, we would expect that *rabx-5* mutations would have little effect on constitutively active RAB-5(Q78L) or inactive RAB-5(S33N). This assumes that there is still guanine exchange activity to load RAB-5 into its GTP bound state, as may be provided by RME-6.

While two different markers cannot be accurately compared in a precise manner, we observed that in a wild type background, the YFP::RAB-5(Q78L) fluorescence pattern looked grossly like the wild type YFP::RAB-5 fluorescence pattern with high intensity in the cell bodies and comparably lower intensity in the dorsal cord. Conversely, the YFP::RAB-5(S33N) fluorescence pattern in the WT background looked similar to the YFP::RAB-5 fluorescence pattern in the *rabx-5* mutant background with cell bodies and the dorsal cord at similar intensities to each other.

**Figure 3:**
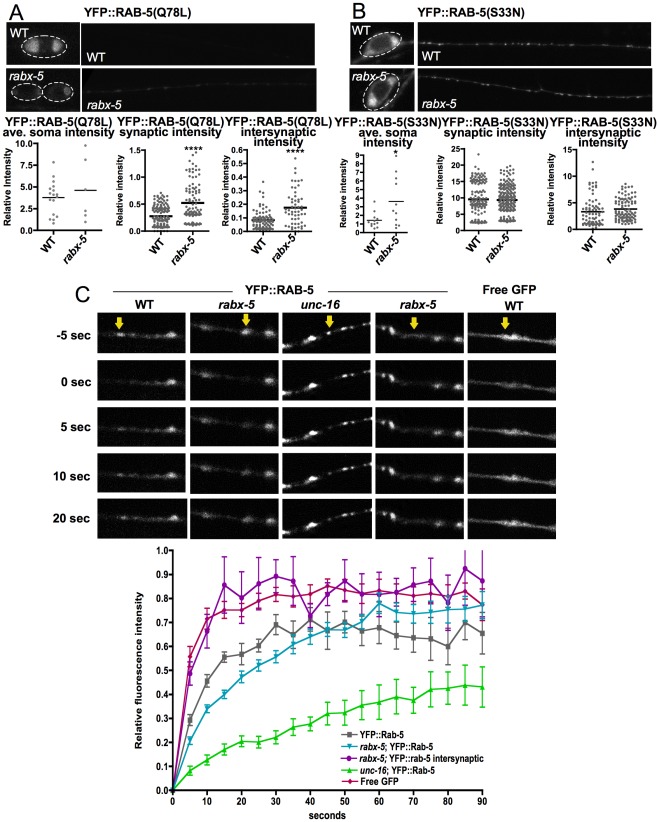
*Rabx-5* acts by biasing the cycling state of RAB-5. A) Soma and dorsal cord of mutant and wildtype GABAergic motor neurons expressing YFP::RAB-5(Q78L). The *rabx-5* mutation does not exhibit as dramatic a phenotype as on YFP::RAB-5. B) Soma and dorsal cord of mutant and wildtype GABAergic motor neurons expressing YFP::RAB-5(S33N). The *rabx-5* mutation does not exhibit as dramatic a phenotype as on YFP::RAB-5. Each point represents a single soma, synaptic puncta, or intersynaptic axonal region, respectively. Bar represents the mean. *p<0.05, ***p<0.001 C) Fluorescent recovery after photobleaching (FRAP) of YFP::RAB-5 in the synaptic regions of WT, the synaptic and intersynaptic regions of *rabx-5,* and the synaptic region of *unc-16* mutant worms in which RAB-5 is biased to the GTP bound state; and FRAP of free GFP. Arrow marks the region of bleaching. Fluorescence recovery after photobleaching of synaptic YFP::RAB-5 in *rabx-5* mutants is significantly slower than WT but faster than *unc-16* mutants (p<0.01 at time points from 5–15 sec; mean ± SEM). Intersynaptic YFP::RAB-5 in *rabx-5* mutant animals exhibits similar dynamics to free GFP.

Comparing YFP::RAB-5(Q78L) intensity between WT and *rabx-5* mutant animals, we see no significant change in cell body intensity and a slight but significant increase in dorsal cord synaptic (WT 0.27±0.02, mutant 0.52±.03 IU) and intersynaptic (WT 0.082±.007, mutant 0.17±.02 IU) intensity (Figure 3A). This increase is not as dramatic as the approximately five-fold increase seen for YFP::RAB-5 intensity. YFP::RAB-5(S33N) exhibits an increase in cell body intensity in *rabx-5* mutants (WT 1.4±0.3 IU, mutant 3.6±0.9 IU), the opposite of the effect on YFP::RAB-5 (Figure 3B). There is no significant change in intensity in the dorsal cord. These results suggest that the effect of the *rabx-5* mutation on RAB-5 protein abundance and localization is at least partially due to a bias in RAB-5 guanine cycling state.

When RAB-5 is in its active GTP-bound state, it is recruited to endosomal membranes; in its inactive, GDP-bound state, it is cytosolic [10], [37]. To describe the cycling state of the YFP::RAB-5 observed in the dorsal cord of WT and *rabx-5* mutant animals, we examined the fluorescence recovery after photobleaching (FRAP) of YFP::RAB-5 (Figure 3C). In WT animals, bleached synaptic YFP::RAB-5 recovered to about 65% of its original intensity and reached a plateau after 60 seconds. In contrast, in *unc-16* mutant animals, in which RAB-5 is biased to its GTP bound state [22], fluorescence recovery is much slower, presumably because it is remaining membrane bound, reaching only 30% recovery after 90 seconds. In *rabx-5* mutant animals, YFP::RAB-5 synaptic fluorescence recovery is slower than wild type but not as slow as in *unc-16* mutants. For example, at 15 seconds, WT recovery has reached 55±2% recovery, whereas *rabx-5* mutants have reached 40±2% recovery (p<0.01) and *unc-16* mutants have reached 17±2% recovery. In comparison, fluorescence recovery is much faster in intersynaptic regions, recovering to 85±3% intensity at 15 seconds. This is a similar recovery rate to what is seen with free GFP alone. WT and *unc-16* intersynaptic fluorescence is too dim for accurate FRAP experiments. We interpret this data to indicate that synaptic YFP::RAB-5 is in its membrane bound state and speculate that its fluorescence recovers slower in *rabx-5* mutants because of slowed RAB-5 cycling. We interpret the dynamics of intersynaptic recovery to indicate that this population is cytosolic, GDP-bound RAB-5 that recovers at the same rate as freely diffusing GFP.

### Specificity of *rabx-5*

In order to distinguish early endosomes from cytosolic YFP::RAB-5 we wished to examine the effects of the *rabx-5* mutation on another marker of early endosomes. We also aimed to determine the specificity of *rabx-5* within the endosomal membrane system. Therefore we examined the effects of the *rabx-5* mutation on a series of endosomal markers. HGRS, an essential tyrosine kinase substrate (HGRS::mCherry), is targeted specifically to early endosomes in a RAB-5 independent manner [38]. Syntaxin-13, a SNARE protein (SYN-13::mCherry), marks both early and recycling endosomes in axonal processes [39] and is present in synaptic vesicles [4]. Rab-11 is present in recycling endosomes, and Rab-7 associates with late endosomes [9], [10].

**Figure 4:**
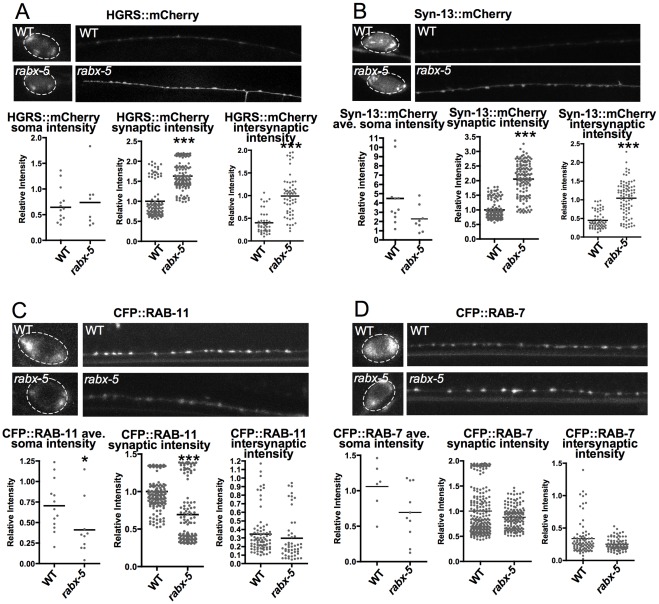
Effects of the *rabx-5* mutation are specific to early endosomal and synaptic compartments. A) Fluorescence intensity of P_unc-25_HGRS::mCherry, a tyrosine kinase substrate targeted specifically to early endosomes, is increased in synaptic and intersynaptic regions in *rabx-5* mutants. B) Fluorescence intensity of P_unc-25_Syntaxin-13::mCherry, a SNARE protein that marks early and recycling endosomes as well as synaptic vesicles is increased in synaptic and intersynaptic regions in *rabx-5* mutants. C) Fluorescence intensity of P_unc-25_CFP::RAB-11 a marker of recycling endosomes is decreased in soma and synaptic regions in *rabx-5* mutants. D) Fluorescence intensity of P_unc-25_CFP::RAB-7, a marker of late endosomes is similar in wild type and in *rabx-5* mutant animals. Each point represents a single soma, synaptic puncta, or intersynaptic axonal region, respectively. Bar represents the mean. *p<0.05, ***p<0.001.

In *rabx-5* mutants, HGRS::mCherry intensity is similar to wildtype within the cell soma but exhibits a significant increase in synaptic (1.63±0.03 IU, 1.0±.04 IU in WT, p<0.0001) and intersynaptic regions (.99±.06 IU, 0.4±.04 IU in WT, p<0.0001) (Figure 4A). A similar pattern is observed in localization of SYN-13::mCherry with significant increases in both synaptic (2.05±.05 IU, 1.00±.03 IU in WT, p<0.0001) and intersynaptic intensity (1.04±.05 IU, 0.45±.03 IU in WT, p<0.0001) (Figure 4B). In contrast, CFP::RAB-11 exhibits decreased synaptic intensity in *rabx-5* mutants (0.69±0.03 IU, 1.00±0.02 IU in WT, p<0.0001) and CFP::RAB-7 exhibits no significant change in intensity from WT (Figure 4C and 4D). We conclude that *rabx-5* regulates the membranous compartments of early endosomes and not simply the cytosolic versus membranous localization of RAB-5. We also observe that *rabx-5* acts primarily on RAB-5 with much less, if any, influence on RAB-11 or RAB-7.

### Role of *rabx-5* on synaptic vesicle populations

**Figure 5:**
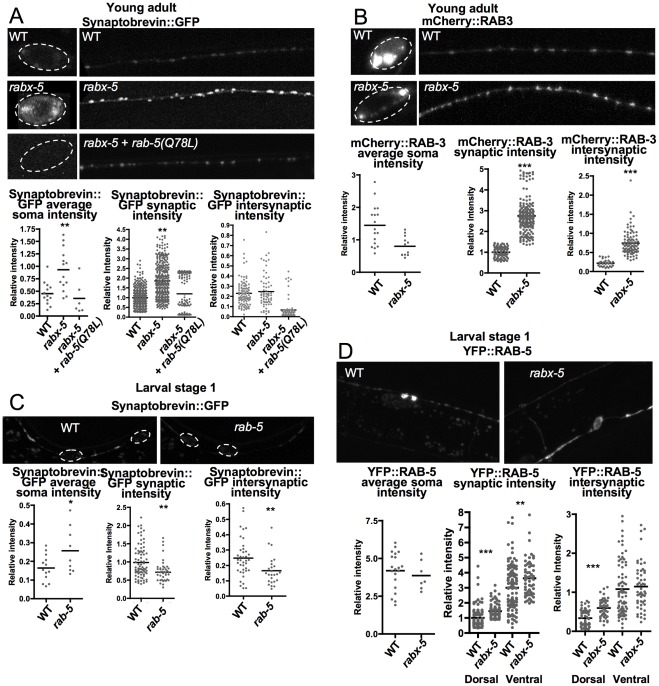
*Rabx-5* mutant animals have aberrant synaptic vesicles. A) P_unc-25_Synaptobrevin::GFP intensity is increased in soma and synaptic regions in *rabx-5* mutant animals. This phenotype is rescued by expression of P_unc-25_mCherry::RAB-5(Q78L). B) P_unc-25_mCherry::RAB-3 intensity is increased in soma and synaptic regions. C) P_unc-25_synaptobrevin::GFP is decreased in soma, synaptic, and intersynaptic regions of larval stage 1 animals. D) YFP::RAB-5 intensity is increased in synaptic regions of the dorsal and ventral cord of larval stage 1 *rabx-5* mutant animals. Each point represents a single soma, synaptic puncta, or intersynaptic axonal region, respectively. Bar represents the mean. *p<0.05, **p<0.01, ***p<0.001.

To examine the effect that altered RABX-5 activity has on synaptic vesicle populations, we examined two proteins associated with synaptic vesicles (Figure 5A and 5B). Synaptobrevin (SNB-1) is a SNARE protein anchored to the membrane of synaptic vesicles and synaptic vesicle precursors [40]. RAB-3 plays a role in synaptic vesicle exocytosis and marks mature synaptic vesicles [41]. Both SNB-1::GFP and RAB-3::mCherry exhibited significantly increased intensity in synaptic puncta of *rabx-5* mutants (1.86±.05 IU, 1.00±.02 IU in WT, p<0.01; 2.75±0.06 IU, 1.00±0.03 IU WT, p<0.0001, respectively), and RAB-3::mCherry had increased intensity in intersynaptic regions as well (0.74±0.03 IU, 0.22±.02 IU in WT p<0.0001). SNB-1::GFP intensity was significantly increased in cell bodies (0.93±0.11 IU, 0.45±0.23 IU in WT, p<0.01) whereas RAB-3::mCherry intensity was significantly decreased (0.80±0.09 IU, 1.45±0.15 IU in WT, p<0.005). Thus, while there are differential effects in cell somas, synaptic vesicles appear to be increased in synaptic regions of *rabx-5* mutants.

To determine if this effect is due to the bias of RAB-5 to its GDP-bound state, we examined SNB-1::GFP fluorescence in animals in which RAB-5(Q78L) was expressed (mCherry::RAB-5(Q78L)). We found that expression of RAB-5(Q78L) rescued the effect of the *rabx-5* mutation on SNB-1::GFP, indicating that *rabx-5* affects synaptic vesicles by biasing the cycling state of RAB-5 (Figure 5A).

To see if mutations directly in RAB-5 also affect synaptic vesicle abundance, we examined SNB-1::GFP in *rab-5* mutants. As these mutants are homozygous lethal beyond larval stage 1 (L1), we examined early L1 animals. At this stage, synaptic vesicles of the DD motor neurons are localized to the ventral cord. In L1 *rab-5* mutant animals, SNB-1 intensity is significantly increased in cell somas (0.26±0.04 IU, 0.16±0.04 IU in WT, p<0.05) and decreased in the ventral cord, in both synaptic (0.76±0.05 IU, 0.99±0.05 IU in WT, p<0.005) and intersynaptic regions (0.17±0.02 IU, 0.25±0.02, p<0.01) (Figure 5C).

We also examined the effect of *rabx-5* mutations on YFP::RAB-5 at the L1 stage and found that they subtly paralleled changes observed in adult animals (Figure 5D). YFP::RAB-5 intensity was increased in both dorsal (1.5±0.05 IU, 1.0±0.06 IU in WT, p<0.0001) and ventral synaptic regions (3.6±0.15 IU, 3.0±0.13 IU in WT, p<0.01) as well as the dorsal cord intersynaptic region (0.60±0.03 IU, 0.34±0.03 IU in WT, p<0.0001) of *rabx-5* mutants.

These data demonstrate that *rabx-5* regulates synaptic vesicles in addition to endosomal compartments. This action may occur through the regulation of RAB-5 as *rab-5* mutant animals also exhibit alterations in localization of the synaptic vesicle marker SNB-1::GFP; however, this altered localization is opposite what one might expect if RABX-5 acted on synaptic vesicles directly through RAB-5. Nonetheless, the effect of *rabx-5* can be rescued by expression of activated RAB-5(Q78L), suggesting the *rabx-5* effect depends on the cycling state of RAB-5.

### Functional effects of *rabx-5* mutations

**Figure 6:**
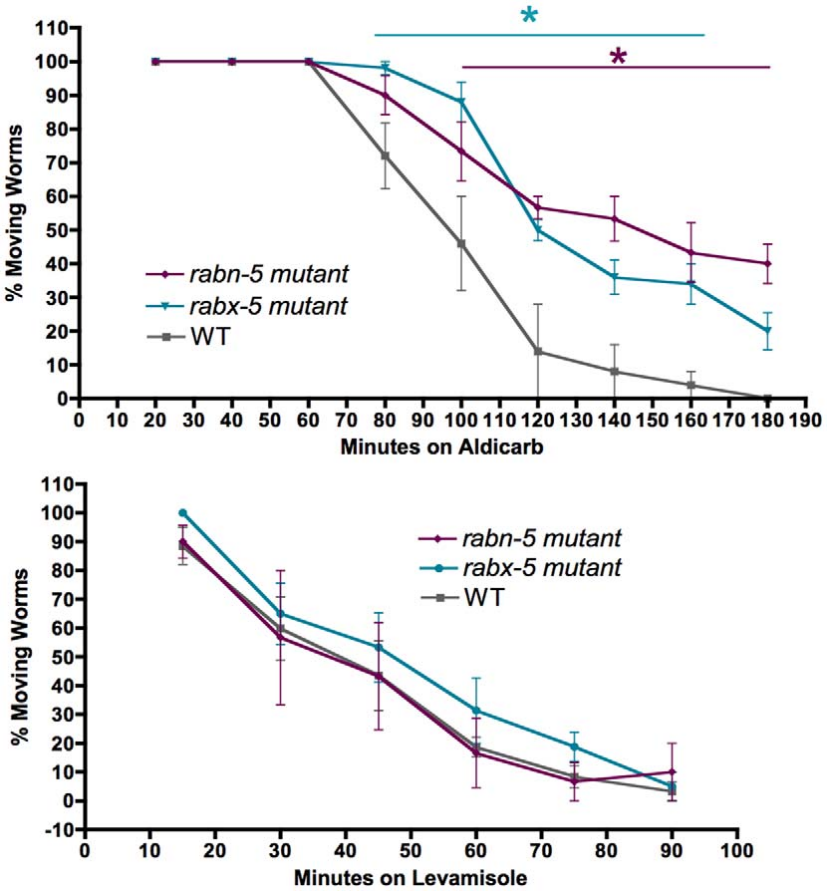
*Rabx-5* and *rabn-5* exhibit subtle presynaptic release defects. Animals placed on 0.5 mM aldicarb, an acetylcholine esterase inhibitor, or levamisole, an acetylcholine receptor antagonist were assayed for movement over time. *Rabx-5* and *rabn-5* mutant animals exhibit significant resistance to aldicarb compared to wild type animals**.** Mean ± SEM, *p<0.05.

Changes in synaptic vesicle marker localization and intensity in *rabx-5* mutants suggest that synaptic function may be altered. However, *rabx-5* mutant animals appear grossly normal. To examine whether the changes in endosomes and synaptic vesicles observed in *rabx-5* mutant animals lead to more subtle synaptic effects, we performed aldicarb and levamisole synaptic function assays (Figure 6). Aldicarb is an acetylcholine esterase inhibitor and levamisole is a cholinergic agonist. Both drugs lead to tetanic paralysis. *Rabx-5* and *rabn-5* mutant worms placed on 0.5 mM aldicarb exhibited significant resistance to aldicarb paralysis compared to control animals. For example, at 120 minutes, only 14% of WT animals are still moving whereas 50% of *rabx-5* mutant animals and 56% of *rabn-5* mutant animals are moving (p<0.0001). The animals did not exhibit any significant difference in paralysis rate when placed on levamisole. These results indicate that *rabx-5* and *rabn-5* mutant animals have a defect in presynaptic neurotransmitter release.

### Role of *rabx-5* in ageing

**Figure 7:**
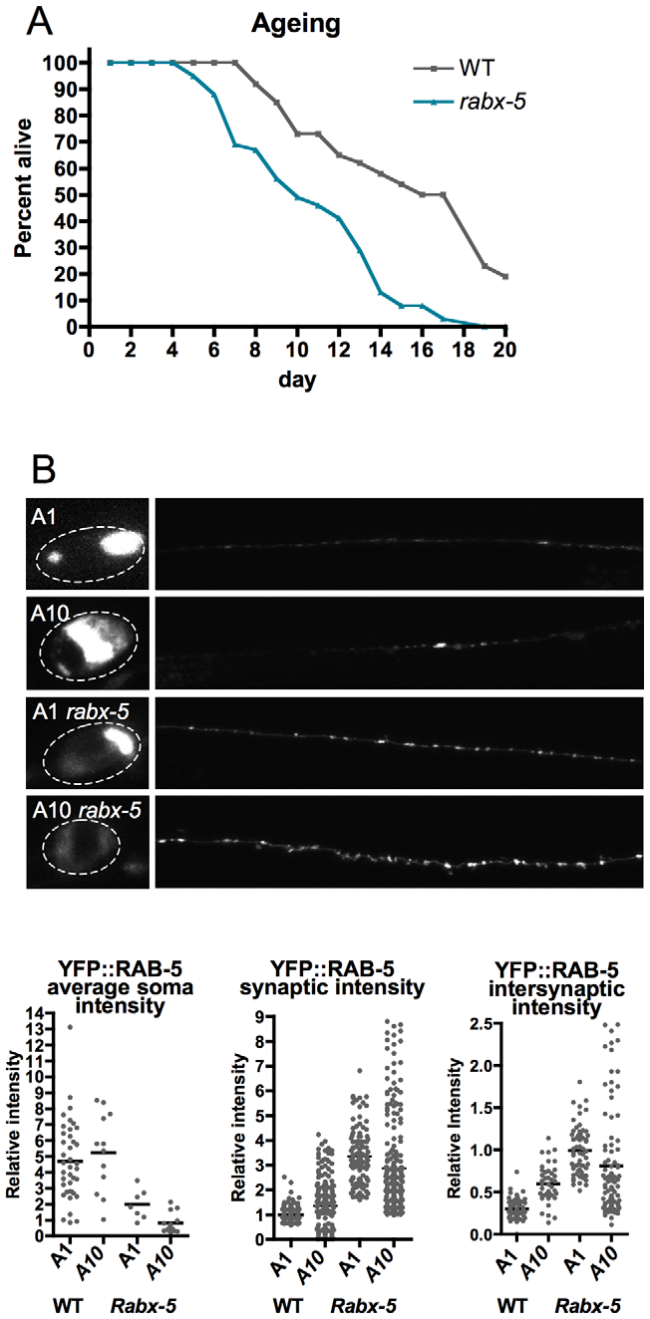
*Rabx-5* mutant animals age faster and RAB-5 endosomal compartments become disorganized with age. A) Survival assay indicates that *rabx-5* mutant animals age faster than wild type animals (log-rank test p<0.0001). B) P_unc-25_YFP::RAB-5 expression in the soma and dorsal cord of adult day 1 and day 10 wild-type and *rabx-5* mutant animals. Synaptic intensity is more variable in adult day 10 animals (F test probability that variances differ p<0.0001). Each point represents a single soma, synaptic puncta, or intersynaptic axonal region, respectively.

Defects in regulation of the endocytic pathway and axonal transport contribute to age-related neurodegenerative disease such as Alzheimer's disease [42], [43], Huntington's disease [44], and fronto-temporal dementia [45]. The Alzheimer amyloid protein precursor is localized to RAB-5 containing organelles of nerve terminals [46], [47], and RAB-5 and APP work together to signal apoptosis [48]. RAB-5 is up-regulated in brains of patients with Alzheimer's disease and Mild Cognitive Impairment [49]. These findings suggest that regulation of RAB-5 may mediate normal and disease state age-related changes in neuronal function. Therefore, we examined whether *rabx-5* mutants exhibited any age-related defects. Compared to WT animals, *rabx-5* mutants aged faster and had a significantly shortened lifespan (log-rank test p<0.0001) (Figure 7A). In *rabx-5* mutant animals, 50% of worms fail to survive past adult day 10, whereas in WT animals, 50% of the worms survive until adult day 16. Synaptic YFP::RAB-5 exhibited more variable intensity in adult day 10 WT worms (0.36±1.07 IU, mean ± SD) than in day 1 adult WT worms (1.00±0.33 IU, F test probability that variances differ p<0.0001), suggesting a disorganization of endosomes with ageing (Figure 7B). This progressive disorganization is also observed in *rabx-5* mutant animals (day 1: 3.35±1.09, day 10: 2.88±1.95, mean ± SD, F test probability that variances differ p<0.0001).

## Discussion

Here we demonstrate that Rabx-5 is an important regulator of RAB-5 endosomal compartments and synaptic vesicles in neurons of *C. elegans.* Previous studies have demonstrated that RAB-5 is an integral member of synaptic vesicles [6], [18], [19] and is needed for maintenance of synaptic vesicle size as well as neurotransmitter release [20], [21], [22], [23]. To understand how RAB-5 is regulated at the synapse, we conducted a forward genetic screen to identify proteins that function alongside of RAB-5 in neurons. We found that one of the isolated mutants, *rabx-5,* regulates synaptic vesicles and synaptic function through its guanine exchange activity on RAB-5.

*Rabx-5* was identified through a new automated screening methodology [25]. Two screens were performed in parallel: an automated screen and a manual screen. Both screens examined approximately 1500 haploid genomes and identified 8–9 mutant alleles. The automated screen had a throughput of 500 haploid genomes per hour whereas we were able to examine approximately 50 haploid genomes per hour with the manual screen. One gene was identified by both screens and the other genes were unique to each screen (as assessed by non-complementation). The automated screen is a significant advance in technology, allowing a 10-fold increase in screening efficiency.

Rabx-5 regulates RAB-5 in both the GABAergic motor neuron soma and synapse, exhibiting a decrease in YFP::RAB-5 in the soma and an increase of YFP::RAB-5 at the synapse. In other systems, RAB-5 is localized to early endosomal compartments and synaptic vesicles [6], [9], [18], [19]. In the soma of *C. elegans* GABAergic motoneurons, RAB-5 localization extends beyond that of SNB-1 [22] suggesting localization to early endosomes as well as synapses. Synaptic vesicle proteins found in the somas of neurons comprise synaptic vesicle and active zone precursors prepared for transport to synaptic terminals [50], [51] and mediate paracrine release of neurotransmitters and neurohormones directly from the soma [52], [53].

RABX-5 functions to promote RAB-5 cycling from the GDP to GTP-bound state via the catalytic vacuolar sorting protein VPS9 domain [30], [31]. The two other *C. elegans* proteins with VPS9 domains, RME-6 and TAG-33 do not exhibit the same YFP::RAB-5 phenotype as *rabx-5*. RME-6 has been shown to function in clathrin coated vesicles in oocytes and coelomocytes [32]. Our data suggest RABX-5 is the primary exchange factor for RAB-5 in neurons with additional activity from RME-6. RAB-5 may have tissue specific exchange factors [30].

We found that *rabn-5* mutant animals exhibit a similar YFP::RAB-5 phenotype as *rabx-5.* Structural evidence suggests that rabaptin functions by preventing the negative autoregulation of rabex-5 [34]. The *rabx-5 rabn-5* synaptic double mutant phenotype is consistent with this model, suggesting that these two genes are working together in the same pathway.

Our data suggest that RABX-5 regulates early endosomal compartments and synaptic vesicles by biasing the cycling state of RAB-5 and by altering early endosomal and synaptic vesicle membrane compartments. YFP::RAB-5 markers locked in a GDP or GTP bound state do not show the same changes in intensity in *rabx-5* mutant animals as freely cycling YFP::RAB-5, suggesting that the phenotype is due at least in part to a bias of the cycling state of RAB-5. The dynamics of RAB-5 FRAP suggest that the RAB-5 that becomes localized in intersynaptic regions in *rabx-5* mutants is cytosolic whereas RAB-5 in synaptic regions is membrane bound. Changes in intensity of two other markers of early endosomes, HGRS-1::mCherry and Syn-13::mCherry, parallel the changes with YFP::RAB-5, indicating that endosomal membrane is altered in addition to the RAB-5 cycling and concomitant membrane-bound state. These changes are not seen in markers of late or recycling endosomes. Synaptic vesicle markers exhibit an increase in synaptic intensity in *rabx-5* mutant animals and this phenotype is rescued by expression of RAB-5(Q78L), suggesting that it depends on the cycling state of RAB-5. Activated GTP-bound RAB-5 promotes the homotypic fusion and enlargement of early endosomes [37]. Conversely activated RAB-5 is required to prevent the homotypic fusion and enlargement of synaptic vesicles and maintain synaptic vesicle size [21]. RAB-5 biased to the GTP-bound state leads to decreased numbers of synaptic vesicles as observed by electron microscope [22]. RAB-5 is present on both synaptic vesicle and endosomal membranes [6], [18], [19]. In *unc-16* mutant *C. elegans* in which RAB-5 is biased to the GTP bound state, there is an decrease in YFP::RAB-5 in intersynaptic axonal regions; and in the *rabx-5* mutant animals, where RAB-5 is biased to the GTP bound state, there is an increase in YFP::RAB-5 in intersynaptic regions. However in both mutants, at the light microscopic level, there is an increase of YFP::RAB-5 at the synaptic regions. We speculate that a bias to the GDP bound state, as in *rabx-5* mutants, leads to increased cytosolic RAB-5 and an increase of RAB-5 containing synaptic vesicle membrane at the synapses whereas a bias to the GTP bound state leads to less cytosolic RAB-5 and increased RAB-5 on the early endosomes of the synaptic region.

We observe subtle effects of the *rabx-5* mutation on synaptic release as measured by aldicarb assays. Resistance to aldicarb paralysis but not levamisole paralysis indicates a decrease in presynaptic release of acetylcholine. This is consistent with work in *Drosophila* demonstrating that impaired RAB-5 function decreases the probability of evoked transmitter release [20], although RAB-5 did not come out positive in an RNAi screen for aldicarb resistance in *C. elegans* [54], perhaps because of the subtlety of the effect.

*Rabx-5* mutant animals age faster than wildtype animals and both wildtype and mutant animals exhibit a disorganization of endosomal compartments with age. The endocytic pathway is implicated in age related neurodegenerative diseases [42], [43]. Patients with Alzheimer's disease have higher levels of RAB-5 in the brain [49]. Amyloid precursor protein and beta-site amyloid cleavage protein (BACE-1) show increasing interactions within endosomes with ageing. A dominant negative RAB-5 inhibits this interaction and decreases Abeta production [55]. The disorganization of the endosomal membrane system may advance these age-associated diseases. By regulating RAB-5 and the early endosomal compartments, *rabx-5* may contribute to healthy ageing.

## Methods

### Screen, Genetics, Plasmids, and Transgenic Animals

*C. elegans* strains were generated from N2(Bristol) and maintained at 20–23 C as described [56]. The manual screen examined ethylmethane sulfonate (EMS) mutagenized *unc-104(e1265)* animals carrying the integrated chromosomal array *P_unc-25_YFP::RAB-5(juIs198)*; *P_ttx-3_RFP.* The automated screen was performed in WT worms carrying the same marker as described [25]. The following mutations were used in this study: *rabx-5(qa7800), rabx-5(tm1512), rabx-5(ok1763), rabn-5(tm1555), rabs-5(ok1513), rme-6(b1014), tag-333(gk431), unc-16(ju146), unc-104(e1265).* Homozygous mutants were confirmed by allele specific PCR and restriction digest. We used the following markers *P_unc-25_YFP::RAB-5(juIs198), P_unc-25_CFP::RABX-5(juEX3160), P_unc-25_YFP::RAB-5(juIs199), P_unc-25_YFP::RAB-5Q78L(juEx1447), P_unc-25_YFP::RAB-5S33N(juEx3411), P_unc-25_mCherry::HGRS-1(juEx3266), P_unc-25_mCherry::SYN-13(juEX3259), P_unc-25_CFP::RAB-11(juEX1145), P_unc-25_CFP::RAB-7(juEx989), P_unc-25_SNB-1::GFP(juIs1), P_unc-25_mCherry::RABX-5Q78L(juEx3904), and P_unc-25_mCherry::RAB-3(juEX1368).* P_unc-25_CFP::RABX-5 (pCZGY1419) plasmid contained rabx-5 cDNA starting from the ATG start site [57]. Plasmids were generated using Gateway technology (Invitrogen, Carlsbad, CA).

### Confocal imaging and analysis

Images were taken on a Zeiss LSM510 laser scanning confocal microscope using a 63X objective. Argon laser output was set to 40% current. Transmission was set to a constant value ranging from 1% to 20% depending on the marker. In the case of YFP::RAB-5, using the same transmission rate across conditions did not capture the full dynamic range of the fluorescence. Control images were taken at each transmission rate used, and intensity was normalized to control as described [22]. Maximum intensity projection merged images from stacks of 5–12 0.4 µm sections were exported as 16 bit tiff files and analysed on Metamorph image analysis software (Molecular Devices, Sunnyvale, CA). Images were thresholded to select individual puncta and average intensity measured for each puncta. Intersynaptic intensity was measured by drawing line scans through the dorsal cord between synaptic puncta. Soma average intensity was measured by drawing a region of interest around the soma. All reported fluorescence intensities had background fluorescence subtracted and were normalized so that average synaptic puncta intensity of that marker in WT animals is equal to one. Data was analyzed using one-way ANOVA followed by Tukey post-test and reported as mean ± SEM except where otherwise noted.

FRAP analysis was performed by bleaching puncta using 100 iterations at 100% laser power over a constant diameter region of interest such that bleaching took 4.9 seconds. Images were captured every 5 seconds at 20% laser transmission. Average intensity of the region of interest was analyzed using Zeiss software, and a background region of interest was subtracted. Intensity was normalized such that intensity before bleaching equaled one and intensity after bleaching equaled zero. Statistical comparisons were made on individual time points using a one-way ANOVA and Bonferonni post-test.

### Pharmacological Assays

Worms were placed on 0.5 mM aldicarb, an acetylcholine esterase inhibitor, or levamisole, an acetylcholine receptor antagonist, and assayed for movement over time. Dishes of at least 10 worms per condition were assayed on at least three different days. Statistical comparisons were made using a two-way ANOVA and Bonferroni post-test.

### Ageing Assay

Animals were grown on standard bacterial plates at 23 C and transferred as needed to distinguish from progeny. Animals were assayed for survival daily and touched with a worm pick to distinguish dead from sluggish worms. At least 27 worms were used per condition. Log-rank test was performed using OASIS software [58].
